# Temporal trend and spatial distribution of cases of mother-to-child transmission of HIV in the state of Santa Catarina, Brazil, 2007-2017: an ecological study

**DOI:** 10.1590/S2237-96222022000100009

**Published:** 2022-07-06

**Authors:** Ilda Vaica Armando Cunga, Bianca Bittencourt, Claudia Maria Augusto da Rosa, Betine Pinto Moehlecke Iser, Gabriel Oscar Cremona Parma, Fabiana Schuelter-Trevisol

**Affiliations:** 1 Universidade do Sul de Santa Catarina, Programa de Pós-Graduação em Ciências da Saúde, Tubarão, SC, Brazil; 2 Governo do Estado de Santa Catarina, Diretoria de Vigilância Epidemiológica, Florianópolis, SC, Brazil

**Keywords:** Time Series Studies, HIV, Acquired Immunodeficiency Syndrome, Seroconversion, Infectious Disease Transmission, Vertical, Residence Characteristics

## Abstract

**Objective:**

To analyze the temporal trend and spatial distribution of mother-to-child HIV transmission in Santa Catarina between 2007 and 2017.

**Methods:**

This was a mixed ecological study with data from the Notifiable Health Conditions Information System. Linear regression was performed for time series analysis and the mean rates in the period and mean annual percentage changes in the rates of HIV-infected pregnant women were calculated, children exposed to HIV during pregnancy, and seroconversion of children exposed to HIV/AIDS during pregnancy, in addition to data geoprocessing.

**Results:**

There were 5,554 records of HIV-infected pregnant women, with a rate of 5.6 pregnant women per 1,000 live births. The mean seroconversion rate was 13.5/100,000 live births (95%CI 6.8;20.1) and it showed a falling trend (APC = -99.4%; 95%CI -99.9;-93.1). The seroconversion rate was more expressive in small towns.

**Conclusion:**

The rate of HIV-infected pregnant women was stable in the period, whereas the number of children infected with HIV through mother-to-child transmission decreased.

## INTRODUCTION

**Table:** 

Study contributions	
Main results	Despite the stable infection rates, we identified a larger number of infected pregnant women and children exposed to risk of mother-to-child HIV transmission, especially in areas of greater population density. There was, however, a reduction in the number of infected children.
Implications for services	The data of this study indicate possible shortcomings in prenatal follow-up and mother and child care, with regard to vertical HIV transmission in municipalities in the state of Santa Catarina.
Perspectives	There is a need for reflection on the strategies used to address the disease and prevention measures that could be better structured in order to achieve the international goal of eradicating mother-to-child HIV transmission.

Infection with the Human Immunodeficiency Virus (HIV), the agent that causes Acquired Immunodeficiency Syndrome (AIDS), is a global public health problem, especially in low-and middle-income countries.^1^ According to the Joint United Nations Programme on HIV/AIDS (UNAIDS), in 2020 more than 37.7 million people of all ages were living with HIV/AIDS worldwide.^1^

Brazilian data have shown that 15,846 (88.8%) cases of AIDS in children under 13 years of age, reported between 2009 and 2020, occurred due to vertical transmission.^3^ Vertical transmission of HIV occurs when the virus is transmitted from mother to child during pregnancy, labor, delivery (contact with cervical-vaginal secretions and maternal blood) or breastfeeding. With regard to monitoring for vertical transmission, the Brazilian Ministry of Health recommends that all pregnant women be screened for HIV infection using rapid tests; in the event of a positive result, they are notified as ‘HIV-positive pregnant women’ and start to follow the treatment and monitoring protocol in order to prevent vertical transmission. When the mother is HIV positive, at birth her child is notified as an ‘HIV-exposed child’ and has follow-up until the outcome of the case is known. Seroconversion results in HIV infection (which defines vertical transmission of HIV), and a new notification is made for epidemiological surveillance purposes.^4^ Specific interventions, such as HIV screening tests and antiretroviral therapy (ART), performed during pregnancy, delivery and postpartum, reduce transmission rates.^4^

In Brazil, the HIV detection rate in pregnant women increased from 2.3 cases/1,000 live births in 2009 to 2.8 cases/1,000 live births in 2019, representing an increase of 21.7%. It is possible that this increase is due to the expansion of HIV testing during prenatal care, child delivery and breastfeeding, resulting in more unknown HIV cases being revealed and, therefore, more case notifications.^3^ Out of the five Brazilian regions, in 2017 the Southern region had the country’s highest detection rate, with 5.8 cases/1,000 live births, and this rate was twice as high as the national rate. Also in 2017 the HIV detection rate in pregnant women in the state of Santa Catarina (in Brazil’s Southern region) was 5.2 cases/1,000 live births.^3^

Considering the importance of early diagnosis of HIV infection and timely treatment of pregnant women in order to minimize seroconversion among exposed children, the increase in notifications is a warning as to the need to pay attention to this group, with a view to adopting the vertical transmission prevention protocol based on knowledge regarding the occurrence of cases of infected pregnant women.

Periodically the Ministry of Health publishes its HIV/AIDS Epidemiological Bulletin, in which it reports HIV detection rates in pregnant women and AIDS detection rates in children under five years of age. Notwithstanding, data on seroconversion among children exposed to HIV during pregnancy are not available. Furthermore, considering that mother-to-child HIV transmission is confirmed by seroconversion, these data are necessary for analysis of mother-to-child HIV transmission, since occurrence of AIDS in children under five years of age can also be attributed to other routes of infection.^3^

There are few studies dedicated to observing seroconversion among children, where these cases are concentrated and whether the rates of vertical HIV infection among children are falling as a consequence of the adoption of intervention measures recommended by clinical protocols and therapeutic guidelines in order to prevent vertical transmission of the virus.^6^

The objective of this study was to analyze the temporal trend and spatial distribution of mother-to-child HIV transmission cases in the state of Santa Catarina, Brazil between 2007 and 2017.

## METHODS

### Design

This was a mixed ecological study, having as its units of analysis the municipalities of the state of Santa Catarina, organized into health macro-regions. We used data on notified cases of HIV-infected pregnant women and children exposed to HIV during pregnancy held on the Notifiable Health Conditions Information System (*Sistema de Informação de Agravos de Notificação* - SINAN) for the period 2007-2017.

### Background

The 2010 demographic census recorded the population of Santa Catarina as being 6,353,055 inhabitants, of whom 1,801,433 were women of childbearing age (15-49 age group). The mean number of live births in the period 2007-2017 was 90,256.^7^ Most of Santa Catarina’s population lives in urban areas (84.0%), with the rural population accounting for the remaining 16.0% of the total. Population density is 65.3 inhabitants/km^2^ and population growth is 1.6% per year. The state has a human development index (HDI) of 0.840 and estimated Family Health Strategy coverage of 78.4%. Geographically, Santa Catarina is formed by 295 municipalities divided between seven health macro-regions: *Sul; Planalto Norte e Nordeste; Meio Oeste; Grande Oeste; Grande Florianópolis; Foz do Rio Itajaí; and Alto Vale do Itajaí*.^8^

### Participants

This study included cases of HIV-infected pregnant women and cases of children exposed to and infected by HIV through mother-to-child transmission based on evidence of seroconversion, recorded on the SINAN system in Santa Catarina in the period 2007-2017.

### Variables

The variables analyzed were: year of notification (between 2007 and 2017); pregnancy outcome (live birth; stillbirth; abortion); year child born; child’s progression (case in progress; infected; not infected; death due to HIV/AIDS; death due to other causes; lost to follow-up; probably not infected; transfer); municipality of residence in Santa Catarina; and Santa Catarina state macro-regions.

The number of HIV-infected pregnant women and the total number of children with perinatal HIV exposure were used to analyze the study outcomes. The dependent variables were the rate of HIV-infected pregnant women, the rate of children exposed to HIV during pregnancy, the seroconversion rate of children exposed to HIV/AIDS during pregnancy (vertical transmission), and the proportion of seroconversion.

### Data source and measurement

This study was based on data held on the Health Ministry’s SINAN, namely data held on HIV-Positive Pregnant Women Investigation Forms and data held on children exposed to HIV during pregnancy, in relation to the state of Santa Catarina.^9^ We included all notification forms dated between January 1^st^ 2007 and December 31^st^ 2017.

The individualized and anonymous databases of children exposed to HIV during pregnancy and HIV+ pregnant woman, from which duplicated records had been removed, were provided by the Santa Catarina State Epidemiological Surveillance Directorate between July and August 2019.

Seroconversion cases were determined by following up children born to HIV-positive mothers, from birth to 18 months of life. A child is considered to be HIV-infected (seroconversion) when two consecutive viral load results above 5,000 copies/ml are obtained.

The rates were calculated taking the reference population to be the number of live births in the state of Santa Catarina between 2007 and 2017, obtained using the TabNet application via the DATASUS website. The indicators analyzed and their calculation methods are described below:

- Rate of HIV-infected pregnant women – number of HIV-infected pregnant women notified in a given year, divided by the total number of live births in the same year, multiplied by 1,000.
- Rate of children exposed to HIV during pregnancy – number of children born alive to HIV-infected women in a given year, divided by the total number of live births in the same year, multiplied by 1,000.
- Seroconversion rate of children exposed to HIV/AIDS during pregnancy – number of children infected with HIV/AIDS or who died from HIV/AIDS in a given year, divided by the total number of live births in the same year, multiplied by 100,000.
- Proportion of seroconversion – number of confirmed mother-to-child HIV/AIDS cases in relation to total children exposed to HIV during pregnancy, multiplied by 100.

Events were mapped using Quantum GIS (QGIS Version 3.22) and Microsoft Excel (2016), as were SINAN tabulated data on notified occurrences. Microsoft Excel was used to build the tables totaling cases by year and by municipality.

We used cartographic data on the municipalities and health regions, retrieved from the official Brazilian cartographic system, in shape format, provided by the Brazilian Institute of Geography and Statistics (IBGE). We also used IBGE population data published in the Official Federal Government Gazette (*Diário Oficial da União*).

Using QGIS, each case was linked according to its municipality code, for the purpose of geoprocessing. The Geographic Information System (GIS) was used to calculate total case incidence per 1,000 or 100,000 inhabitants, depending on the indicator, for the period from 2007 to 2017.

### Statistical methods

Analysis was performed using Microsoft Office Excel and SPSS v.21 (IBM, Armonk, New York, USA). Descriptive analysis was performed for the purpose of data presentation. Generalized linear regression (Prais-Winsten model) with robust variance was performed to analyze the time series of rates of HIV-infected pregnant women, rates of children exposed to HIV during pregnancy and seroconversion rates for the study period. The Durbin Watson statistic was used to check for autocorrelation, with values close to 2 expected to be indicative of absence of serial autocorrelation.

The response variables (Yi) were the respective rates, while the explanatory variable (Xi) was the year of notification. Statistical associations with a p-value < 0.05 were considered to be significant. Thus, a falling trend was considered to be when the p-value was < 0.05 and the regression coefficient was negative; while a rising trend was considered to be when the p-value was < 0.05 and the regression coefficient was positive. The mean rates for the period 2007-2017 and the mean annual percent change (APC) of the rates were calculated, using the values obtained in the regression analysis according to the method proposed by Antunes & Cardoso,^11^ with a 95% confidence interval (95%CI).

With regard to geoprocessing, the variables were mapped thematically, with representation of classes according to Jenks Natural Breaks classification on the map with the greatest distribution, which was kept on the other maps in order to enable comparisons and analysis of trends of the phenomenon. This method is appropriate for mapping values that are not uniformly distributed, as is the case of the phenomenon studied in this work.

### Ethical aspects

The study project was approved by the *Universidade do Sul de Santa Catarina* Research Ethics Committee, as per Opinion No. 3.137.377, issued on February 8^th^ 2019.

## RESULTS

In Santa Catarina between 2007 and 2017, 5,554 HIV-infected pregnant women and 4,559 children exposed to HIV during pregnancy were notified. Considering the total number of live births in the study period, the mean rate of HIV-infected pregnant women was 5.6/1,000 live births, the mean rate of children exposed to HIV during pregnancy was 4.6/1,000 live births, and the mean seroconversion rate was 13.5 HIV-infected children/100,000 live births.

Table 1 shows the distribution of reported HIV cases among pregnant women and children exposed to HIV during pregnancy, as well as the seroconversion rate. In the period from 2007 to 2017, the lowest HIV infection rate among pregnant women was 5.2 cases/1,000 live births in 2007, while the highest was 6.0 cases/1,000 live births in 2015. The number of live births to pregnant women infected with HIV during pregnancy varied greatly throughout the period studied (Table 1).

**Table 1:** Distribution of infected pregnant women, children exposed to HIVa during pregnancy and seroconversion rate of children exposed to HIV/AIDSb during pregnancy, among live births with follow-up, Santa Catarina, 2007-2017

Year of notification	HIV+ pregnant women (n)	Live births born to HIV+ pregnant women (n)	Live births (n)	Rate of HIV-infected pregnant women/1,000 live births^a^	Rate of children exposed to HIV/1,000 live births^b^	Seroconversion^c^		Seroconversion rate/100,000 live births^d^
						n	%	
2007	428	355	82,530	5.2	4.3	17	4.8	20.6
2008	470	512	85,744	5.5	6.0	26	5.1	30.3
2009	490	492	84,010	5.8	5.9	7	1.4	8.3
2010	476	546	85,091	5.6	6.4	14	2.6	16.5
2011	509	413	87,975	5.8	4.7	25	6.1	28.4
2012	475	398	89,295	5.3	4.5	14	3.5	15.7
2013	477	362	90,547	5.3	4.0	8	2.2	8.8
2014	540	395	94,049	5.7	4.2	5	1.3	5.3
2015	585	395	98,192	6.0	4.0	11	2.8	11.2
2016	558	424	96,159	5.8	4.4	1	0.2	1.0
2017	536	267	99,222	5.4	2.7	2	0.7	2.0
**2007-2017**	**5,554**	**4,559**	**992,814**	**5.6**	**4.6**	**130**	**2.9**	**13.5**

a) HIV: Human Immunodeficiency Virus; b) AIDS: Acquired Immunodeficiency Syndrome. c) Number of HIV-infected pregnant women notified in the year, divided by total live births in the same year, multiplied by 1,000. d) Number of live births born to HIV-infected pregnant women in the year, divided by total live births that year, multiplied by 1,000. e) Number of confirmed HIV/AIDS cases via vertical transmission (n) in relation to total children exposed to HIV during pregnancy, multiplied by 100 (%). f) Number of HIV-infected children or children who died from HIV/AIDS, divided by total live births that year, multiplied by 100,000.

Seroconversion also varied greatly during the period, while mean seroconversion was 2.9%. The seroconversion rate in children exposed to HIV/AIDS during pregnancy was 20.6/100,000 live births in 2007 and 2.0/100,000 in 2017. In the period analyzed, the highest HIV seroconversion rates were found in 2008 (30.3/100,000 live births) and in 2011 (28.4/100,000 live births), while the lowest rate was found in 2016 (1.0/100,000 live births) (Table 1).

**Figure 1:**
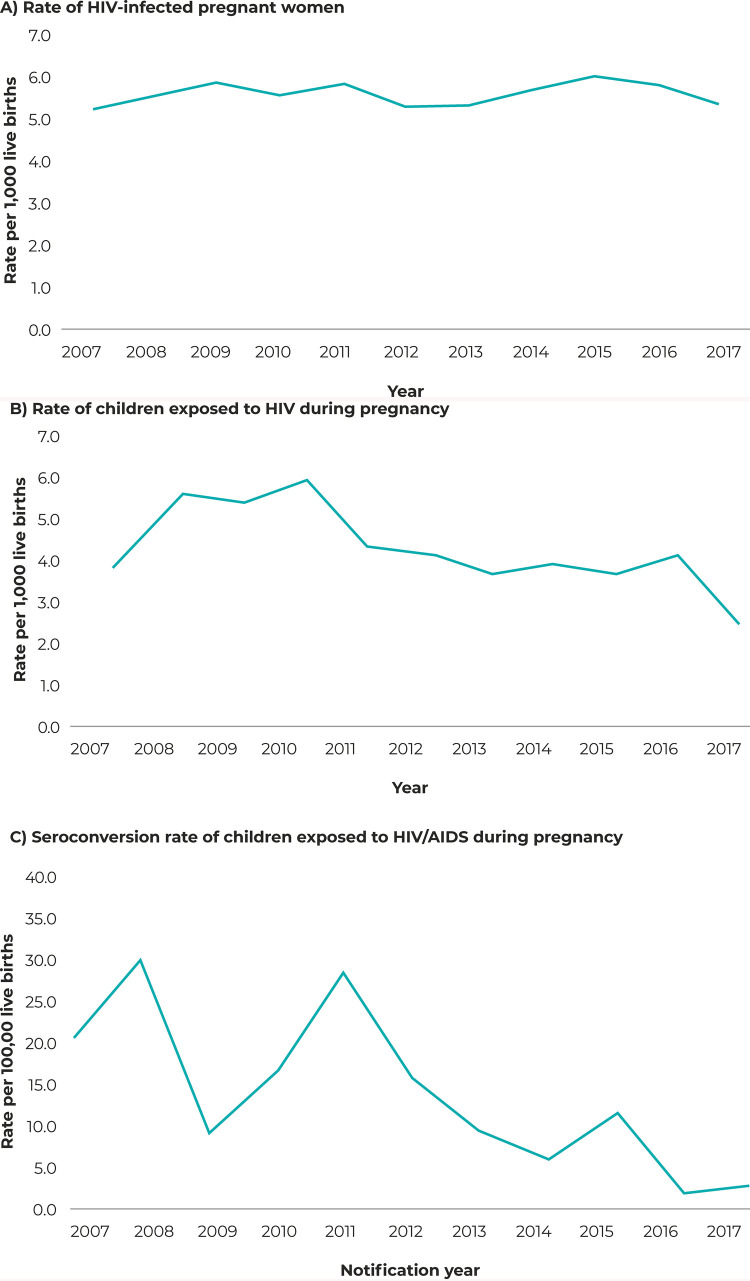
Temporal trend of the rate of HIV-infected pregnant women (A), rate of children exposed to HIV during pregnancy (B) and seroconversion rate of children exposed to HIV/AIDS during pregnancy (C), Santa Catarina, 2007-2017

The mean rate of children exposed to HIV during pregnancy was 4.6 children/1,000 live births (95%CI 3.9;5.4), and the trend was stable in the period analyzed (APC = -38.3; 95%CI -69.1;21.1) (Table 2 and Figure 1B). The mean seroconversion rate was 13.5/100,000 live births (95%CI 6.8;20.1), with a mean annual reduction of 99.4% (95%CI -99.9;-93.1), as shown in Table 2 and Figure 1C. The rate of HIV-infected pregnant women and children with perinatal exposure to HIV in Santa Catarina was high, although it remained stable (Figure 1A and 1B).

**Table 2:** Temporal trend of rates of HIV-infecteda pregnant women, children exposed to HIV during pregnancy and seroconversion of children exposed to HIV/AIDSb during pregnancy, Santa Catarina, 2007-2017

Rates	Mean (95%CI^c^)	Beta coefficient (95%CI)	p-value	R^2 d^ (%)	APC^e^	Trend^f^
Rate of HIV-infected pregnant women^g^	5.6 (5.4;5.8)	0.02 (-0.05;0.09)	0.528	63.9	0.00 (-10.9;23.0)	→
Rate of children exposed to HIV during pregnancy^g^	4.6 (3.9;5.4)	-0.21 (-0.51;0.08)	0.139	38.0	-38.3 (-69.1;21.1)	→
Seroconversion rate of children exposed to HIV/AIDS during pregnancy^h^	13.5 (6.8;20.1)	-2.25 (-3.34;-1.16)	0.001	63.5	-99.4 (-99.9;-93.1)	↓

a) HIV: Human Immunodeficiency Virus; b) Aids: Acquired Immunodeficiency Syndrome; c) 95%CI: 95% confidence interval; d) R2: coefficient of determination; e) APC: Annual Percent Change; f) Trend: stable (→), rising (↑), falling (↓); g) Per 1,000 live births; h) Per 100,000 live births.

**Figure 2:**
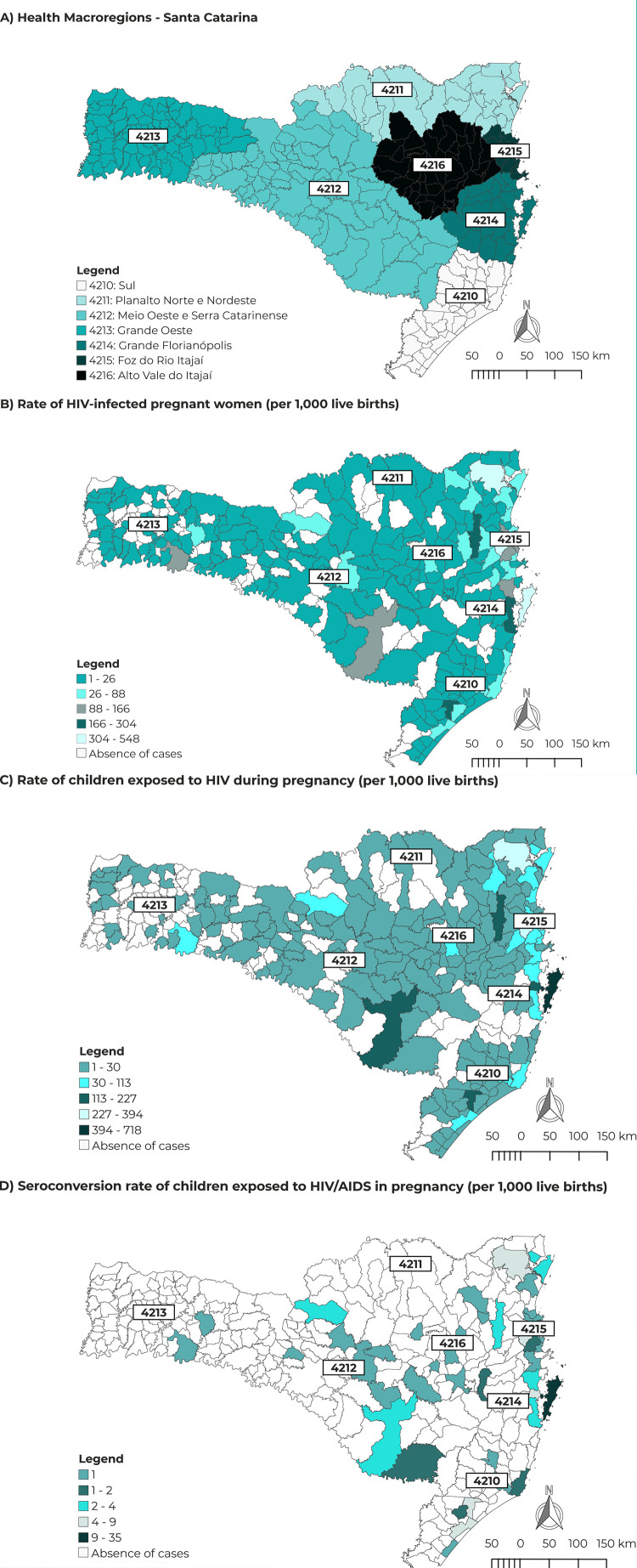
Spatial distribution of HIV cases per municipality of residence among pregnant women and children exposed to and infected with HIV, Santa Catarina, 2007-2017 A) Santa Catarina Macro-Regions; B) Distribution of the rate of HIV-infected pregnant women; C) Distribution of the rate of children exposed to HIV during pregnancy; e D) Distribution of the seroconversion rate of children exposed to HIV/AIDS during pregnancy

Figure 2 shows the number of cases of infections related to mother-to-child HIV transmission in children, in the different health regions of the state, in the period from 2007 to 2017. The *Foz do Rio Itajaí* health region had the highest rates of HIV-infected pregnant women, represented by the municipalities of Itajaí (13.4/1,000 live births) and Camboriú (12.7/1,000 live births) (Figure 2B). The *Grande Florianópolis* health region had the highest rate of children exposed to and infected by HIV during pregnancy (Figure 2C), especially the state capital Florianópolis (11.6/1,000 live births) and the city of Leoberto Leal (452.5/100,000 live births) (Figure 2D).

## DISCUSSION

This study analyzed the distribution of cases of seroconversion of children exposed to HIV/AIDS during pregnancy in the state of Santa Catarina between 2007 and 2017. The analysis of the mother-to-child transmission temporal trend found stability in the rate of HIV-infected pregnant women and in the rate of children exposed to HIV over the period, with a reduction in the child seroconversion rate. The highest number of cases of exposed pregnant women and children was concentrated in the coastal region; however, the highest seroconversion rates did not follow the same distribution and was more expressive in small cities. Although the rate of infected pregnant women remained stable, it was twice as high as the national average, and is considered a risk factor for mother-to-child HIV transmission.

This study found a rate of 5.6 HIV-infected pregnant women per 1,000 live births, this being twice as high as the national rate of 2.8 per 1,000 live births in the same period.^3^ The trend found in the period analyzed revealed that the increase in the number of notifications of cases of HIV infection in pregnant women in Santa Catarina was not accompanied by an increase in the number of seroconversion cases, suggesting that timely detection and adequate treatment of these cases can prevent perinatal transmission of HIV to children.

The reduction in seroconversion rates found in Santa Catarina can be compared with the reduction in the HIV/AIDS rate in children under five years of age in Brazil as a whole, found in 2019, when the national average was 1.9 case per 100,000 inhabitants.^3^ As such, recommended interventions, such as prenatal care and follow-up, use of ART with virological and immunological monitoring, indication of cesarean delivery when maternal viral load is above 1,000 copies/ml, use of oral antiretroviral drugs in neonates, and formula feeding, have had a great impact in reducing mother-to-child HIV transmission in Brazil,^4^ especially in the Southern region where the detection rates of HIV-infected pregnant women are higher.

Early diagnosis of infection and use of antiretroviral therapy have enabled viral suppression and CD4+ T lymphocyte levels to be maintained, thus making HIV/AIDS a chronic condition and with greater survival time and better quality of life for infected people.^14^ These measures enable HIV-infected women to become pregnant without there necessarily being intrauterine exposure to the virus, as long as clinical protocols are properly followed.^15^ It is noteworthy, however, that greater occurrence of HIV-infected pregnant women increases the exposure of children to the risk of mother-to-child HIV transmission, especially when HIV infection is diagnosed when women are already pregnant, during prenatal care and child delivery.^16^ The detection rate of pregnant women with HIV in Brazil has been increasing in recent years, due to the increase and expansion of prenatal diagnosis, given the ease of access to rapid testing.^17^

The analysis of the spatial distribution of cases indicated that both HIV infection in pregnant women and children infected with HIV were concentrated in coastal regions, with higher population density and generally better developed in social and cultural terms. Previous studies have shown that the largest numbers of cases of people living with HIV are found in municipalities with a high HDI, in developing countries, with a high degree of urbanization and with more than 100,000 inhabitants.^19^ Another study conducted in Santa Catarina found higher rates of infection among pregnant women in the *Foz do Rio Itajaí* and *Grande Florianópolis* health regions, located in the coastal area of the state, with major tourist attractions and Brazil’s third largest seaport.^21^ It is noteworthy that smaller municipalities had a higher seroconversion rate, suggesting late diagnosis and shortcomings in prenatal care regarding the adoption of prophylactic measures against mother-to-child HIV transmission.^22^ Miranda et al. found several flaws in the “cascade” of care of HIV-positive pregnant women, showing that sometimes women’s and children’s health care services may be disconnected from each other.^12^

Between 2007 and June 2018, 247,795 cases of HIV infection were reported in Brazil, 116,292 of which related to infected pregnant women, around 30% of which were reported in the country’s Southern region.^3^ When diagnosis of HIV infection occurs during pregnancy, there is less time between initiation of treatment and childbirth to achieve viral suppression and prevent mother-to-child transmission.^25^

This study found a discrepancy in the SINAN databases between the number of infected pregnant women and the number of children exposed to HIV during pregnancy. In principle, these numbers should be similar. However, even considering cases of abortion and stillbirth, there was still a difference of 2% of live births with no record of exposure to HIV during pregnancy on the HIV-exposed children’s database in comparison to the records of HIV-infected pregnant women. This difference may indicate computer system flaws, duplicated records of HIV-infected pregnant women or delay in notification, or even cases of underreporting, which may influence the data analysis.^28^

Among the limitations of the study, we highlight the use of secondary databases, in which there are information gaps for some variables resulting from unknown data. Another point to be emphasized is that of no linkage between records held on the two SINAN databases, i.e. the HIV-infected pregnant women database and the children exposed to HIV during pregnancy database. Linkage was not possible because the names were removed from both databases to ensure anonymity, thus preventing pairing of mother-child cases which would have assisted with temporal progression and detection of missing cases. Moreover, annual adequacy between the number of pregnant women and the number of live and infected births in the same period could be checked, since a full-term pregnancy lasts approximately 40 weeks, and newborns can have follow-up until they ate 18 months old in order to monitor for HIV seroconversion. Consulting the Mortality Information System (*Sistema de Informações sobre Mortalidade* - SIM), the National Lymphocyte Count Network Laboratory Test Control System (*Sistema de Controle de Exames Laboratoriais da Rede Nacional de Contagem de Linfócitos* - SISCEL) and the Antiretroviral Medication Logistics Control System (*Sistema de Controle Logístico de Medicamentos Antirretrovirais* - SICLOM) could elucidate factors associated with mother-to-child transmission, in the sense of a more complete analysis of risk factors, such as adherence to antiretroviral treatment, initiation of treatment, virological and immunological control, and analysis of deaths resulting from HIV/AIDS infection.

The conclusion is reached that there is an increasing number of infected pregnant women, which implies more children exposed to the risk of mother-to-child HIV transmission, with concentration in urban areas with higher population density. Although the seroconversion rate is declining, oscillations were observed during the period analyzed. It is extremely important to carry out studies aimed at investigating avoidable risk factors. The results of this study can be useful for informing the debate on the pattern of the HIV/AIDS epidemic in Santa Catarina, indicating shortcomings in prenatal care and assistance for mothers and children in all the state’s municipalities. Evidence was provided of the need to reflect on the strategies used to address the disease and adoption of better structured prevention measures, in order to achieve the international goal of eradicating mother-to-child transmission of the human immunodeficiency virus.^29^
